# Presence of Essential Hypertension or Diabetes Mellitus Is a Predictor of Intracranial Bleeding in Elderly Patients: A Study of 108 Patients with Isolated Thrombocytopenia from a Single Reference Center

**DOI:** 10.4274/tjh.2013.0161

**Published:** 2015-05-08

**Authors:** Rajan Kapoor, Hara Prasad Pati, Manoranjan Mahapatra, Anuradha Monga

**Affiliations:** 1 All India Institute of Medical Sciences (AIIMS), Department of Hematology, New Delhi, India; 2 MD India Healthcare Services (TPA), New Delhi, India

**Keywords:** Thrombocytopenia, Elderly, Immune thrombocytopenic purpura, Intracranial bleeding

## Abstract

**Objective:**

Thrombocytopenia poses a significant problem in the elderly. Not only are there varied causes, but it is also associated with significant morbidity and mortality. We carried out a study to learn the causes of isolated thrombocytopenia in elderly patients and to correlate the severity of thrombocytopenia and bleeding manifestations with various etiologic factors and comorbidities.

**Materials and Methods:**

A total of 108 patients above 50 years of age presenting with isolated thrombocytopenia (platelet counts of <100x109/L with normal hemoglobin and total leukocyte counts) were enrolled in the study. Detailed history and clinical examinations were carried out for each patient. Complete blood counts were analyzed by automated cell counter. Peripheral smears were examined in all cases. HbsAg, anti-HCV, and anti-HIV testing by enzyme-linked immunosorbent assay was done in all patients. Wherever clinically indicated, bone marrow aspiration biopsy and cytogenetic studies were done.

**Results:**

Out of 108 patients, 102 (94.4%) presented with bleeding tendencies. Twenty-nine (26.8%) presented with serious (World Health Organization grade 3/4) bleedings. Major findings were immune thrombocytopenic purpura in 79 (73.1%), myelodysplastic syndrome in 7 (6.5%), drug-induced thrombocytopenia in 7 (6.5%), and connective tissue disorder in 4 (3.7%) cases. Ten patients presented with intracranial bleedings. Upon logistic regression analysis, comorbidities in the form of essential hypertension and diabetes mellitus were significantly associated with occurrence of intracranial bleeding. There was no correlation of serious bleedings with platelet counts.

**Conclusion:**

Isolated thrombocytopenia in the elderly is associated with significant morbidity. Diligent clinical and laboratory evaluation is required to elucidate the cause of thrombocytopenia in the elderly. Comorbidities in this population are associated with serious bleedings and not low platelet counts as is commonly thought.

## Abstract (tr)

**Amaç:**

Trombositopeni ileri yaşlarda ciddi sorunlar doğurur. Bunda sadece çeşitli sebeplerin olduğunu söylemek yeterli olmayacaktır zira önemli hastalıklarla ve ölümle de çokça ilişkilendirilir. İleri yaştaki hastalarda görülen izole trombositopeninin sebeplerini öğrenmek ve trombositopeninin şiddetiyle çeşitli etiyolojik faktörler ve beraberinde görülen ek hastalıklardaki kanamalar arasında var olan ilişkiyi göstermek amacıyla bir çalışma yürüttük.

**Gereç ve Yöntemler:**

İzole trombositopeni (normal hemoglobin ve toplam lökosit sayısı mevcut, trombosit sayısı <100x109/L) görülen 50 yaşın üstündeki toplam 108 hasta bu çalışmaya dahil edildi. Her hasta için ayrı ayrı geçmiş sorgulaması ve klinik incelemeler yapıldı. Tam kan sayımı otomatik hücre sayıcısında analiz edildi. Her olguda çevresel kan yayması incelendi. Her hastada Eliza yöntemi ile HbsAg, anti HCV ve anti HIV testleri yapıldı. Klinik olarak belirtilen her noktada kemik iliği aspirasyon biyopsisi ve sitogenetik analizler yapıldı.

**Bulgular:**

Yüz sekiz hastadan 102’sinde (%94,4) kanama eğilimi görüldü. Yirmi dokuz hastada (%26,8) ciddi kanamalar (Dünya Sağlık Örgütü 3./4. derece) yaşandı. Elde edilen ana bulgulara göre 79 olguda (%73,1) immün trombositopenik purpura, 7’sinde (%6,5) miyelodisplastik sendrom, 7’sinde (%6,5) ilaca bağlı oluşan trombositopeni ve 4’ünde de (%3,7) bağ dokusu hastalığı görüldü. Hastaların 10’unda intrakranial kanamalar yaşandı. Lojistik regresyon analizine göre esansiyel hipertansiyon ve diabetes mellitus gibi eşlik eden hastalıkların, intrakranial kanamalarla önemli ölçüde ilişkilendirildiği gösterilmiştir. Trombosit değerleriyle ciddi kanamalar arasında herhangi bir ilişki bulunamamıştır.

**Sonuç:**

İleri yaşlardaki hastalarda görülen izole trombositopeni ciddi morbidite nedenidir. Yaşlı hastalarda görülen trombositopeninin sebebini açıklamak için özenli klinik ve laboratuvar değerlendirmelerin yapılması gerekmektedir. Bu popülasyonda görülen diğer hastalıklar ciddi kanamalarla ilişkilidir, ancak genel kanının aksine düşük trombosit değerleriyle kanama arasında herhangi bir ilişkisi yoktur.

## INTRODUCTION

Thrombocytopenia poses an especially significant problem in the elderly, where it can not only lead to life-threatening bleedings, but may also be a presenting feature of underlying hematologic malignancies, which are more common in this age group [1]. Thrombocytopenia in the elderly is associated with significant morbidity and mortality, leading to frequent hospitalizations [2]. The most common type of isolated thrombocytopenia, immune thrombocytopenic purpura (ITP), is not uncommon in the elderly. In a study of 245 adult patients, the highest age-specific incidence was found in patients above 60 years of age [3]. Compared to patients of less than 40 years of age, those older than 60 have been reported to have a higher incidence of major hemorrhagic complications of ITP and a higher ITP-related mortality [1,4]. In view of potential comorbidities such as hypertension, gastrointestinal disorders, and exposure to antiplatelet medications, older individuals have a greater risk of bleeding than children [1]. In addition to ITP, myelodysplastic syndrome (MDS) may present as isolated thrombocytopenia in the elderly [5]. However, no large-scale study of isolated thrombocytopenia in the elderly and its effect on morbidity and mortality has been reported. To analyze the clinical profile, etiology, and correlation of severity of bleeding with various etiologic factors and comorbidities in patients of more than 50 years of age with isolated thrombocytopenia, we carried out a study at a tertiary care referral center.

## MATERIALS AND METHODS

### Patients and Methods

All patients above the age of 50 years presenting with isolated thrombocytopenia to the outpatient department or admitted to the hospital with isolated thrombocytopenia were enrolled in the study. This study was an observational, both prospective and retrospective, single institution-based study. All studied patients had applied to the All India Institute of Medical Sciences for treatment. The study was given clearance by the ethics committee of the All India Institute of Medical Sciences, New Delhi. For the purpose of the study, isolated thrombocytopenia was defined as a platelet count of less than 100x109/L with normal total leucocyte count and hemoglobin concentration. Low platelet counts as counted by automated cell counter were confirmed by peripheral smear (PS) examination and any pseudothrombocytopenia was ruled out. Patients with low platelet counts (<100x109/L) by automated cell counter but normal counts by PS were excluded from the study. 

A detailed history and examination was recorded for all enrolled patients. Particular attention was paid to the presence and severity of bleeding symptoms, site of bleeding, and duration of illness prior to presentation. Bleeding severity was graded according to the World Health Organization (WHO) bleeding scale [6]. Detailed history regarding drug intake, including alternative medicines, was recorded. History regarding comorbidities that are prevalent in this age group, mainly hypertension, diabetes mellitus, and coronary artery disease, was also recorded. Physical examination findings of each patient were noted. The presence of bleeding symptoms and their severity was assessed. A detailed systemic evaluation, which included abdominal, respiratory, cardiac, and neurologic examination, was also carried out. Complete blood counts were analyzed by automated cell counter (Sysmex). HbsAg, anti-HCV, and anti-HIV testing by enzyme-linked immunosorbent assay was done in all patients. Thyroid function tests were done in all patients. Bone marrow aspiration and biopsy were done in the following cases:

a) Abnormal findings on clinical evaluation, especially presence of lymphadenopathy and/or organomegaly.

b) Abnormal findings on PS in any of the cell lines.

c) Strong clinical suspicion of MDS or ITP. Bone marrow cytogenetics was also studied in cases of suspected MDS. In the presence of clinical pointers toward connective tissue disorder, especially in females, special tests like ANA, dsDNA, lupus anticoagulant, and anticardiolipin antibodies were also done. 

### Diagnosis

#### Immune Thrombocytopenic Purpura

For the diagnosis of ITP, absence of any obvious initiating and/or underlying cause of thrombocytopenia was mandatory. Diagnosis of ITP was based on recently published diagnostic guidelines [7]. All categories of ITP (acute, persistent, and chronic) were included in the study. Bone marrow aspirate and biopsy were done in all cases. Fulfilment of the following criteria were essential for the diagnosis:

1- Normal PS examination, except isolated thrombocytopenia. Large platelets were supportive of but not mandatory for diagnosis. 

2- Normal or increased megakaryocyte number on visual assessment by a hematopathologist on bone marrow biopsy. 

3- Absence of any megakaryocyte dysplasia, which is indicative of myelodysplasia.

4- Absence of clinically apparent associated conditions or causes of thrombocytopenia.

#### Myelodysplastic Syndrome

MDS was diagnosed on the basis of the WHO criteria [8], which require the presence of dysplasia in hematopoietic cell series or the presence of cytogenetic abnormalities typical of MDS.

#### Drug-Induced Thrombocytopenia

Drugs were implicated as a cause of isolated thrombocytopenia if there was definite prior history of ingestion of a drug known to cause thrombocytopenia along with resolution of thrombocytopenia upon discontinuation of the drug. 

#### Statistical Analysis

Statistical software R 2.11.1 was used (http://www.R-project.org). Logistic regression analysis was carried out to study correlations of binary outcomes with different variables.

## RESULTS

A total of 108 patients above 50 years of age were enrolled in the study. The median age was 55 years. The oldest patient was 81 years old. Fifty-six patients were female and 52 were male. Mean duration of illness prior to presentation was 140.2 days, with a range from 12 days to 224 days. According to dietary habits, 57 patients (52.7%) were vegans while the rest consumed a mixed diet. Age-wise distribution is illustrated in Table 1. 

One hundred and two patients had bleeding as a presenting feature. Six patients were incidentally found to have thrombocytopenia during evaluation of unrelated illness. Mucocutaneous bleedings in the form of petechiae and ecchymoses were the only symptom in 38.8% (42/108) of patients. Ear, nose, and throat (ENT) bleedings in the form of gum bleedings or epistaxis were the second most common presentation with 13.8% (15/108) of patients presenting with some form of ENT bleeding. Among the severe bleedings, gastrointestinal bleeding was the presenting feature in 13.8% (15/108) of patients. Ten patients (9.2%) presented with intracranial (IC) bleedings. Out of these, 7 were subdural bleedings, while 3 patients had intraparenchymal bleedings. The underlying diagnosis was ITP in 9 of these patients, while 1 patient had drug-induced thrombocytopenia. On grading the severity of bleeding according to the WHO bleeding scale, 63 patients (58.3%) had grade 1, while serious bleedings of grades 3 and 4 were seen in 17 (15.7%) and 12 (11.1%) patients, respectively. Essential hypertension was the most common comorbidity in the study population with 41 (38%) patients having a history of hypertension. Type 2 diabetes mellitus was present in 27 (25%) patients. Primary hypothyroidism was present in 17.5% (19/108) of patients. Median platelet count was 22x109/L, with a range from 2x109/L to 80x109/L. Breakdown of the final diagnoses of these 108 patients is given in Table 2.

Occurrence of intracranial (IC) bleeding in patients of isolated thrombocytopenia and its correlation with other variables were analysed (Table 3). Baseline platelet count had no significant effect on occurrence of IC bleedings. Presence of essential hypertension and diabetes mellitus had a significant association with occurrence of IC bleedings. Patients with arterial hypertension were 8.33 times more likely to have IC bleeding (95% CI: 1.64-50). Similarly, diabetics were 3.7 times more likely to have IC bleeding (95% CI: 1.04-14.3). Increased age (p=0.892), male sex (OR: 2.04, 95% CI: 0.55-7.69), and presence of ITP (OR: 3.91, 95% CI: 0.48-32.1) were not associated with occurrence of IC bleedings. On analysis of severe (WHO grade 3/4) bleeding, essential hypertension was associated with 3.09 times higher risk (95% CI: 1.25-7.66). Patients with ITP had 4.17 times more association with severe bleeding (95% CI: 1.15-14.28). There was no significant difference in baseline platelet count among different diagnostic categories.

## DISCUSSION

A total of 108 patients above the age of 50 years presenting with isolated thrombocytopenia were analyzed in this study. We decided to take 50 years as the cut-off for the definition of “elderly” as this age better represents our elderly population. Although there are many definitions of old age, there is no general agreement on the age at which a person becomes old. In a recent study of prevalence of MDS in an elderly population in Africa, the WHO took as a cut-off 50 years [9]. The most common underlying diagnosis in this age group was ITP, which was expected as the highest age-specific incidence of ITP has been reported in patients above 60 years of age [3]. Although most patients with ITP have overall good outcomes, excessive morbidity and mortality can be attributed to both bleeding and the immunosuppressive treatments to which these patients are subjected [5]. In our series, 29 patients (26.8%) presented with severe (WHO grade 3/4) bleedings. Ten patients (9.2%) had IC bleeding. Out of these, 9 had ITP, whereas in 1 patient, thrombocytopenia was induced by drugs. In a study of 47 elderly patients with ITP, the incidence of brain hemorrhage was 3/47 (6.3%) [6], which is less than the rate of 9/79 (11.3%) found in our study. Although none of the patients died due to thrombocytopenic bleedings, the incidence of IC bleedings is much higher than that reported in younger patients. The most common comorbidities were primary hypertension and type II diabetes mellitus, which were seen in 37.9% and 25% cases, respectively. Though comorbidities in ITP have been reported in the past, none of them were specifically studied in the elderly [10]. The importance of this lies in the fact that occurrence of IC bleeding is significantly associated with the presence of comorbidities. Patients with arterial hypertension were 8.33 times more likely to have IC bleeding (95% CI: 1.64-50). Patients with diabetes mellitus were 3.7 times more likely to have IC bleeding (95% CI: 1.04-14.3). Similarly, essential hypertension was associated with 3.09 times greater risk of having severe grade III/IV bleedings (95% CI: 1.25-7.66). Patients with ITP had 4.17 times greater risk of severe bleedings (95% CI: 1.15-14.28) in comparison to patients with thrombocytopenia due to other etiologies. Baseline platelet count was not predictive of serious bleedings in this elderly population. To our knowledge, the detrimental effect of comorbidities specifically in the elderly population has not been reported. A study of 178 elderly patients with ITP did not report any serious bleeding complications attributable to low platelet counts [11]. In the largest reported retrospective study of ITP in the elderly, risk of severe bleeding was associated with both platelet count (p<0.001; OR: 0.973) and age (p=0.025; OR: 1.039) [12]. On the contrary, according to the results of our study, the presence of comorbidities was a predictor of serious bleedings in elderly patients with ITP.

## CONCLUSION

Isolated thrombocytopenia is a common clinical problem in the elderly and is associated with significant morbidity and mortality. In patients with ITP, the presence of essential hypertension and diabetes mellitus, which are common in this age group, is associated with occurrence of IC bleeding, which leads to significant morbidity in this otherwise benign disease. Due to the retrospective nature of this study, we could not analyze any correlation between glycemic or blood pressure control and the presence of IC bleedings in this population. Another weakness of this study was that we could not assess B12 and folic acid status in our patients, which are known to be affected by dietary habits, as half of our patients were vegans. Optimal management of comorbidities in this age group is of paramount importance if we are to prevent serious bleedings in the elderly thrombocytopenic population. 

## Figures and Tables

**Table 1:**
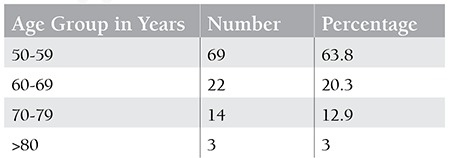
Age-wise distribution of patients with isolated thrombocytopenia.

**Table 2:**
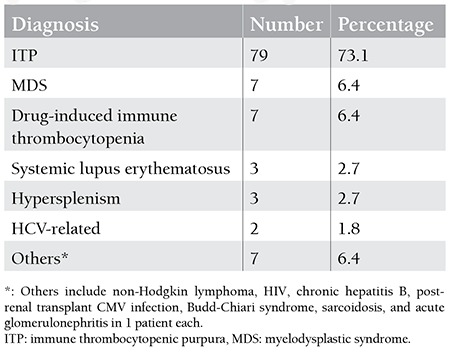
Diagnosis in 108 patients above 50 years of age presenting with isolated thrombocytopenia.

**Table 3:**
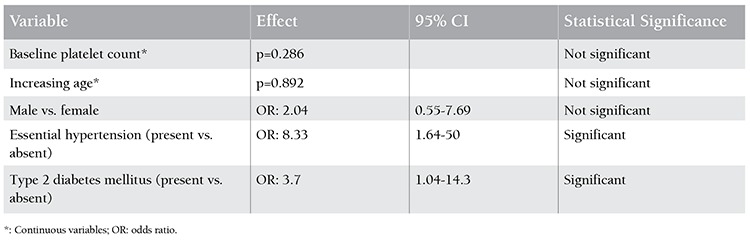
Correlation between occurrence of intracranial (IC) bleeding and other variables by logistic regression analysis; n=10.
